# Barriers facing emergency physicians in providing urgent care to pediatric patients in Saudi Arabia – a cross-sectional study

**DOI:** 10.25122/jml-2024-0291

**Published:** 2024-12

**Authors:** Housam Almadani, Malik Almailabi, Marwan Henaidi, Mohammed Almelibari, Yazeed Almhgadi, Hawazeni Alsulaimani, Moayed Almanabri

**Affiliations:** 1 Department of Pediatric Hematology/Oncology, King Fahad Armed Forces Hospital, Jeddah, Kingdom of Saudi Arabia; 2 Department of Emergency Medicine, King Faisal Specialist Hospital & Research Center, Jeddah, Kingdom of Saudi Arabia; 3 Department of Pediatric Hematology/Oncology, King Abdullah Children Specialty Hospital, Jeddah, Kingdom of Saudi Arabia; 4 Ibn Sina National College for Medical Studies, Jeddah, Kingdom of Saudi Arabia; 5 Faculty of Medicine, Umm Al-Qura University, Makkah, Kingdom of Saudi Arabia; 6 King Fahad Armed Forces Hospital, Jeddah, Kingdom of Saudi Arabia

**Keywords:** pediatric patient, emergency physician, confidence

## Abstract

Efforts to improve healthcare services have been ongoing, particularly in equipping emergency departments (EDs) to handle pediatric cases. However, many EDs continue to lack specialized equipment and adequately trained personnel, exposing children to significant health risks. This study aimed to identify self-reported barriers among emergency physicians in managing pediatric patients and assess their confidence levels in pediatric care. A cross-sectional survey was conducted between March 2023 and January 2024 among emergency physicians dealing with pediatric emergencies practicing in the Kingdom of Saudi Arabia. Data were collected through an online self-administered questionnaire, which included demographic details, perceived barriers in pediatric care, availability of pediatric services, recommendations for improvement, and a 4-item confidence assessment. Out of 214 ED physicians, 197 responded (response rate: 92.1%), with junior residents comprising 40.1% of participants. The most reported barriers were determining accurate medication doses (20.8%) and managing interactions with parents (19.8%). Confidence in pediatric care was reported by 46.2% of participants and was significantly higher among consultants (AOR = 2.522; 95% CI, 1.187–5.358; *P* = 0.016) and those who encountered pediatric patients regularly during their shifts (AOR = 3.113; 95% CI, 1.396–6.946; *P* = 0.006). Conversely, lower confidence was observed among physicians who recommended workshops and mandatory training courses for improving pediatric care quality (AOR = 0.475; 95% CI, 0.228–0.988; *P* = 0.046). The findings highlight a lack of confidence among ED physicians in managing pediatric patients, with confidence levels varying based on the age of patients and frequency of pediatric exposure. The most common challenges were accurate medication dosing, interactions with parents, and addressing a diverse range of pediatric diseases. Consultants and regular exposure to pediatric cases were associated with increased confidence, while a perceived need for workshops and mandatory courses predicted decreased confidence.

## INTRODUCTION

Enhancing emergency services, especially for children, is one of the main objectives of the World Health Assembly through the Sustainable Development Goals (SDG) and Millennium Development Goals (MDG) [1]. In 2019, 7.4 million children and teenagers died worldwide, mostly from treatable or avoidable causes. Additionally, developing nations have a greater burden of child mortality, with the greatest under-five death rate (74 per 1000 live births), more than nine times higher than that of Europe (8 per 1000 live births). From 93 deaths per 1000 live births in 1990 to 38 deaths per 1000 live births in 2019, the global under-five mortality rate has dropped by 60% [2].

Although general emergency departments often lack specialized equipment and trained personnel to manage pediatric emergencies, they still attend to many sick children [3,4]. This shortage of specialized pediatric emergency physicians leaves children as a particularly vulnerable population receiving emergency care from healthcare professionals who may not be adequately prepared [5].

Few studies have explored the self-confidence of emergency medicine (EM) physicians in managing pediatric cases across the region. Therefore, this study’s findings provide a valuable contribution to the existing literature, highlighting the critical role of physician confidence in delivering effective care to pediatric patients in emergency settings [6,7].

Data from the 2018 National Hospital Ambulatory Medical Care Survey showed 130 million ED visits yearly, with children under 15 accounting for 25.6 million [1]. Nevertheless, most general emergency physicians do not receive subspecialty training in pediatric emergency medicine, nor are they often required to complete mandatory courses or additional training to manage pediatric emergencies [8].

Due to the global shortage of fellowship-trained pediatric emergency physicians, care for pediatric emergencies is frequently provided by general emergency physicians or general pediatricians. According to a study done in 2019, 57% of physicians working in non-pediatric EDs were not comfortable examining, diagnosing, or treating pediatric patients, particularly younger children and those critically ill [9]. Similarly, a 2018 study assessing community ED staff found significant discomfort among healthcare providers in managing pediatric resuscitations, largely attributed to the rarity of such cases in their clinical settings [10].

The Saudi Board of Emergency Medicine (SBEM) incorporates mandatory training in pediatric emergency medicine within its residency program. Trainees are required to complete a four-week pediatric emergency department rotation during each junior year, which doubles to eight weeks during the senior years, under the supervision of fellowship-trained pediatric emergency physicians. Additionally, the program mandates a four-week pediatric intensive care unit rotation [11]. Despite this structured training, board-certified emergency physicians may encounter long periods—sometimes months or years—without managing pediatric emergencies. This variability arises from differences among hospitals in the frequency and volume of pediatric cases managed by general emergency physicians, further underscoring the challenges in maintaining pediatric-specific expertise in general emergency practice.

The care provided to the pediatric population by general emergency physicians is crucial and should be assessed and improved. National and international studies should be conducted to assess and improve pediatric emergency care provided by non-specialized practitioners. These studies should assess the current state of pediatric emergency care and identify specific gaps where targeted training and preparation can be implemented efficiently and cost-effectively.

To date, no national study has evaluated emergency physicians' comfort and confidence level in providing urgent care to pediatric patients. This study aims to address this gap by assessing the confidence and preparedness of general emergency physicians in managing pediatric emergencies. Additionally, it seeks to highlight key areas for improvement. The findings of this research will serve as a foundation for future studies and quality improvement initiatives aimed at establishing and maintaining high standards of care in pediatric emergencies. This study's primary objective was to assess emergency physicians' self-reported level of comfort and confidence in caring for pediatric patients. The secondary objective was to identify key areas for improvement to enhance the care delivered to pediatric patients by general emergency physicians.

## MATERIAL AND METHODS

### Study design and location

This study was an observational cross-sectional study. Data collection occurred between March 2023 and January 2024 under the auspices of the Armed Forces Scientific Research Center. A sample of 214 emergency physicians practicing in the Kingdom of Saudi Arabia were included in the study. A validated and reliable self-reported questionnaire was utilized for data collection.

### Inclusion and exclusion criteria

The study included all adult emergency physicians in Saudi Arabia who managed pediatric patients during the study period. Emergency physicians who were on leave, either internally or externally, at the time of the study were excluded.

### Sample size calculation

The target population comprised all emergency physicians employed by the Saudi Ministry of Health during the study period, estimated to be around 1000 individuals. The required sample size was calculated using the Raosoft software (http://www.raosoft.com/samplesize.html) at a 90% confidence level with an assumed 50% response distribution and a margin of error of ± 5%. The minimum required sample size was determined to be 214 participants.

### Data collection methods and instruments

Data were collected using a self-administered questionnaire in English distributed electronically via Google Forms. The questionnaire link was disseminated through WhatsApp groups for emergency physicians, with multiple reminders sent over one month to maximize the response rate. The questionnaire comprised basic demographic data, barriers when dealing with pediatric patients, availability of pediatric services, recommendations for improvement, and a 4-item questionnaire to measure confidence when caring for pediatric patients.

### Survey validation

The questionnaire design was informed by a previously validated tool developed by Alofi and Bakarman [12]. Informed consent was obtained as part of the first question in the survey. The questionnaire included sections on demographic characteristics, facility support, comfort levels in managing different age groups, and suggestions for improvement. To ensure the validity of the questionnaire, a pilot test was conducted with ten physicians and minor adjustments were made based on their feedback. Data from the pilot test were excluded from the final analysis.

### Scoring

Physicians’ confidence in managing pediatric patients was assessed using a 4-item questionnaire, with each item coded as either 'comfortable' (1) or 'not comfortable' (0). The total confidence score was calculated by summing the responses across all items, with a score ranging from 0 to 4 points. The greater the score, the greater the confidence. Physicians were considered confident if the score was 3 to 4 points and not confident if the score was less than 3 points.

### Statistical analysis

The data were analyzed using the Statistical Packages for Software Sciences (SPSS) version 26 (Armonk, New York, IBM Corporation, USA). Descriptive statistics were presented as numbers and percentages (%) for all categorical variables and as the mean and standard deviation for continuous variables. The Chi-square test was used to evaluate the relationship between confidence levels and socio-demographic characteristics, as well as recommendations for improving pediatric services. Based on the significant results, a multivariate regression analysis was subsequently performed to determine the significant independent predictors of confidence when managing pediatric patients. Values were considered significant with a *P* value of less than 0.05.

## RESULTS

197 out of 214 ED physicians responded to our survey (response rate: 92.1%). Among the respondents, 58.9% practiced in Jeddah, with junior residents constituting the largest professional group (40.1%). In addition, 78.7% reported having at least less than 5 years of working experience (Table 1).

**Table 1:** Socio-demographic characteristics of emergency physicians (*n* = 197)

Variable	*n* (%)
City of practice	
∙ Jeddah	116 (58.9%)
∙ Mecca	27 (13.7%)
∙ Riyadh	24 (12.2%)
∙ Taif	05 (02.5%)
∙ Medinah	06 (03.0%)
∙ Abha	05 (02.5%)
∙ Jubail	05 (02.5%)
∙ Others	09 (04.6%)
Qualification	
∙ ER Associate Consultant	20 (10.2%)
∙ ER Consultant	20 (10.2%)
∙ ER Senior Resident	78 (39.6%)
∙ ER Junior Resident	79 (40.1%)
Years of experience	
∙ <5 years	155 (78.7%)
∙ 5 – 10 years	32 (16.2%)
∙ 11 – 15 years	04 (02.0%)
∙ >15 years	06 (03.0%)

### Barriers when dealing with pediatric patients

**Figure 1:**
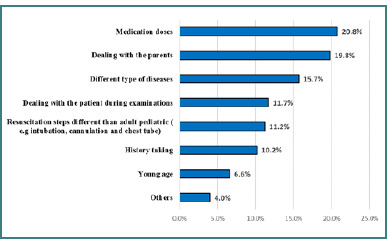
Barriers when dealing with pediatric patients

The most common barrier when dealing with pediatric patients was medication doses (20.8%), followed by dealing with the parents (19.8%) and the diversity of pediatric diseases (15.7%) (Figure 1).

### Availability of emergency pediatric services and recommendations for improvement

29.4% indicated that a pediatric emergency medicine (PEM) consultant was always available during their shift, and 52.4% stated that pediatric resuscitation beds were always available. In addition, 58.4% said that pediatric services are always available at their institution, and 40.1% reported that the basic equipment for pediatric resuscitation needed in the ER was always available. Only 9.6% reported always seeing pediatric patient during their shifts. Recommendations for more training in pediatrics, workshops and mandatory courses, equipment and pediatric resuscitation beds, and access to pediatric resources and management guidelines were raised by 68%, 78.2%, 74.6%, and 88.8% of participants, respectively (Table 2).

**Table 2:** Availability of emergency pediatric services and recommendations for improvement (*n* = 197)

Variable	*n* (%)
Do you have PEM consultant available for all your shifts?	
∙ Never	50 (25.4%)
∙ Rarely	10 (05.1%)
∙ Sometimes	41 (20.8%)
∙ Often	38 (19.3%)
∙ Always	58 (29.4%)
Do you have a pediatric resuscitation bed in your ER?	
∙ Never	34 (17.3%)
∙ Rarely	12 (06.1%)
∙ Sometimes	26 (13.2%)
∙ Often	22 (11.2%)
∙ Always	103 (52.3%)
Do you have pediatric services in your institution?	
∙ Never	30 (15.2%)
∙ Rarely	17 (08.6%)
∙ Sometimes	21 (10.7%)
∙ Often	14 (07.1%)
∙ Always	115 (58.4%)
Do you have the basic equipment for pediatric resuscitation that you need in your ER?	
∙ Never	07 (03.6%)
∙ Rarely	27 (13.7%)
∙ Sometimes	42 (21.3%)
∙ Often	42 (21.3%)
∙ Always	79 (40.1%)
How often do you see pediatric patients during your shift?	
∙ Never	14 (07.1%)
∙ Rarely	58 (29.4%)
∙ Sometimes	81 (41.1%)
∙ Often	25 (12.7%)
∙ Always	19 (09.6%)
More training in pediatrics during residency	
∙ Yes	134 (68.0%)
∙ No	63 (32.0%)
Workshops and mandatory courses	
∙ Yes	154 (78.2%)
∙ No	43 (21.8%)
Equipment and pediatric resuscitation beds	
∙ Yes	147 (74.6%)
∙ No	50 (25.4%)
Access to pediatric resources and management guidelines	
∙ Yes	175 (88.8%)
∙ No	22 (11.2%)

### Physicians' confidence in caring for pediatric patients

**Figure 2:**
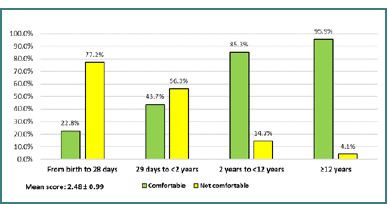
The level of confidence when caring for pediatric patients

**Figure 3:**
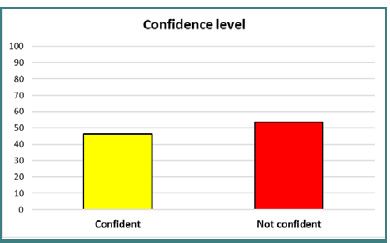
Overall confidence levels of emergency physicians when managing pediatric patients

Physicians had more confidence when caring for a patient aged 12 years or older (95.9%), with the lowest confidence noted in managing neonates. The overall mean confidence score was 2.45 (SD = 0.99) (Figure 2). Additionally, 46.2% of emergency physicians expressed confidence in managing pediatric patients, while 53.8% indicated they were not confident (Figure 3).

### Factors influencing physician confidence

When examining the relationship between confidence levels and socio-demographic characteristics as well as recommendations for improving pediatric services, it was found that confidence in managing pediatric patients was significantly higher among consultants (*P* = 0.005), physicians who did not recommend workshops or mandatory courses (*P* = 0.009), and those who frequently or always cared for pediatric patients during their shifts (*P* = 0.009) (Table 3).

**Table 3:** Relationship between physician confidence and socio-demographic characteristics (*n* = 197)

Variable	Level of confidence		*P*-value
	Confident n (%) (*n* = 91)	Not confident n (%) (*n* = 106)	
City of practice			
∙ Inside Jeddah	58 (63.7%)	58 (54.7%)	0.200
∙ Outside Jeddah	33 (36.3%)	48 (45.3%)	
Qualification			
∙ Resident	65 (71.4%)	92 (86.8%)	0.008 **
∙ Consultant	26 (28.6%)	14 (13.2%)	
Years of experience			
∙ <5 years	69 (75.8%)	86 (81.1%)	0.364
∙ ≥5 years	22 (24.2%)	20 (18.9%)	
How often do you see pediatric patients in your shift?			
∙ Never/Rarely	23 (25.3%)	49 (46.2%)	0.009 **
∙ Sometimes	43 (47.3%)	38 (35.8%)	
∙ Often/Always	25 (27.5%)	19 (17.9%)	
More training in pediatrics during residency			
∙ Yes	58 (63.7%)	76 (71.7%)	0.232
∙ No	33 (36.3%)	30 (28.3%)	
Workshops and mandatory courses			
∙ Yes	65 (71.4%)	89 (84.0%)	0.034 **
∙ No	26 (28.6%)	17 (16.0%)	
Equipment and pediatric resuscitation beds			
∙ Yes	65 (71.4%)	82 (77.4%)	0.340
∙ No	26 (28.6%)	24 (22.6%)	
Access to pediatric resources and management guidelines			
∙ Yes	78 (85.7%)	97 (91.5%)	0.198
∙ No	13 (14.3%)	09 (08.5%)	

*P* value was calculated using the chi-square test

** Significant at P < 0.05 level.

Multivariate regression analysis revealed that being a consultant and occasionally seeing pediatric patients during their shifts were independent significant predictors of increased confidence, while the recommendation of workshops and mandatory courses was the independent significant predictor of decreased confidence. This further indicates that compared to residents, consultants were 2.5 times more likely to demonstrate confidence when caring for pediatric patients (AOR = 2.522; 95% CI, 1.187–5.358; *P* = 0.016). Physicians who sometimes see pediatric patients during their shifts were 3.1 times more likely to have confidence when managing pediatric patients than those who never/rarely see pediatric patients (AOR = 3.113; 95% CI, 1.396–6.946; *P* = 0.006). On the contrary, compared to physicians who did not recommend workshops and mandatory courses, physicians who recommended workshops and mandatory courses had decreased confidence levels by at least 52% (AOR = 0.475; 95% CI, 0.228–0.988; *P* = 0.046) (Table 4).

**Table 4:** Multivariate regression analysis of factors influencing physician confidence in managing pediatric patients (*n* = 197)

Factor	AOR	95% CI	*P* value
Qualification			
∙ Resident	Ref		
∙ Consultant	2.522	1.187 – 5.358	0.016 **
How often do you see pediatric patients in your shift?			
∙ Never/Rarely	Ref		
∙ Sometimes	3.113	1.396 – 6.946	0.006 **
∙ Often/Always	1.347	0.624 – 2.905	0.448
Workshops and mandatory courses			
∙ Yes	0.475	0.228 – 0.988	0.046 **
∙ No	Ref		

AOR, Adjusted Odds Ratio; CI, Confidence Interval.

** Significant at *P* < 0.05 level.

## DISCUSSION

This study aimed to determine the confidence of emergency physicians when providing immediate care to pediatric patients. The results of this study showed that the confidence levels of emergency physicians when managing pediatric cases were not ideal. Based on our criteria, 53.8% lacked confidence in providing care, while 43.2% were likely confident in handling pediatric cases. This is consistent with the study done in Taiwan [13].

Among the 258 emergency physicians, 52.3% were somewhat confident in managing newborns, infants, and clinical procedures. They also reported that physicians' overall satisfaction was associated with learning experience in pediatric wards and ED rotations. However, the lack of exposure to pediatric cases made learners less confident. Contradicting these reports, Langham *et al*. [14] documented that emergency physicians were completely prepared to manage pediatric cardiopulmonary arrests, while Goldman *et al*. [10] observed high comfort levels among providers in caring for injured and acutely ill children. It is necessary to improve the confidence of emergency physicians when caring for children who need urgent care. In this scenario, educational and training programs could significantly improve confidence in providing care for pediatrics who require immediate care.

### Significant factors of confidence

Being a consultant and seeing pediatric patients more often were the independent significant predictors of increased confidence. However, resident physicians, those who had never/rarely seen pediatric patients, and those who attended workshops and courses were associated with lower confidence. There are many factors attributed to the lack of confidence among physicians, including lack of training and experience and lack of exposure to pediatric patients. This is almost comparable to the study of Fawcett *et al*. [15].

Factors that increased physicians' comfort level include seeing more patients over time, being adept in treatment procedures, and real-time support provided by general emergency physicians. This corroborates the reports of Slubowski *et al*. [16]. Emergency physicians' competency in specific procedures depended on training background, the number of visits, and years of experience.

### Confidence in managing specific pediatric age groups

Regarding confidence when caring for pediatric patients, our results indicate that increasing confidence was associated with increasing pediatric age. For instance, emergency physicians were comfortable managing pediatric cases aged 2 to <12 years (85.3%), which increased to 95.9% managing cases aged 12 years or older. On the contrary, 77.2% of our respondents were uncomfortable caring for newborn babies (from birth to 28 days). Among American providers, 50% were comfortable managing patients at least 3 months of age [9]. However, resident physicians in Taiwan showed low confidence in caring for newborns and infants [13].

### Recommendations for improvement

The survey revealed a range of recommendations to improve pediatric urgent care among emergency physicians. Recommendations such as access to pediatric resources and management guidelines (88.8%), workshops and mandatory courses (78.2%), equipment and pediatric resuscitation beds (74.6%), and more training in pediatrics during residency (68%) were identified as key areas for improvement. Incidentally, we noted that physicians who recommended additional training and courses were more likely to have low confidence in managing pediatric cases. Hence, such recommendations are important to increase their confidence levels.

### Confidence barriers

It is necessary to discuss the barriers hindering confidence among our physicians. Medication doses (20.8%), dealing with parents (19.8%), and different types of diseases (15.7%) were the most prominent deterring factors encountered by our respondents. In the USA, [9] the most commonly mentioned barriers were a lack of access to journals or pediatric experts, time constraints, and low institutional priority instead of low pediatric volume. This corroborates the study done in Canada [17]. Key barriers include time constraints, lack of mental health clinician support, and hesitation in the pediatric ED physicians' role. However, in another study in the USA [10], three major obstacles emerged, including the emotional toll of caring for a sick child, the knowledge and skill limitations attributed to the infrequency of training and actual clinical events, and the acknowledgment of pediatric-specific quality and safety deficiencies.

### Study limitations

The findings of this study are subject to some limitations. First, the sample size of 197 participants may not be sufficient to generalize findings to the broader population of emergency physicians. Second, the convenience sampling method could result in sampling bias and lead to a lack of diversity in the target population. Third, key demographic variables, including age and physician gender, were not collected, and thus, we cannot measure gender and age differences in relation to the level of confidence. Lastly, a cross-sectional survey could be prone to bias, unable to determine cause and effect, and cannot be used to measure behavior over time.

## CONCLUSION

There was a lack of confidence among emergency physicians when caring for pediatric patients. Consultants who frequently saw pediatric patients during their shifts tended to be more comfortable caring for pediatric patients than the rest of the physicians. As a potential solution, our respondents proposed a thoughtful solution for improvement, including more training in pediatrics during residency, workshops, courses, equipment, and access to pediatric resources and management guidelines. Hence, considering these points is necessary to improve national pediatric emergency readiness.

## Data Availability

Further data is available from the corresponding author upon reasonable request.
